# A Phylogenetic Re-Analysis of Groupers with Applications for Ciguatera Fish Poisoning

**DOI:** 10.1371/journal.pone.0098198

**Published:** 2014-08-05

**Authors:** Charlotte Schoelinck, Damien D. Hinsinger, Agnès Dettaï, Corinne Cruaud, Jean-Lou Justine

**Affiliations:** 1 UMR 7138 “Systématique, Adaptation, Évolution”, Muséum National d'Histoire Naturelle, Département Systématique et Évolution, Paris, France; 2 Fisheries and Oceans Canada, Molecular biology, Aquatic animal health, Moncton, Canada; 3 Génoscope, Centre National de Séquençage, Évry, France; Northwest Fisheries Science Center, NOAA Fisheries, United States of America

## Abstract

**Background:**

Ciguatera fish poisoning (CFP) is a significant public health problem due to dinoflagellates. It is responsible for one of the highest reported incidence of seafood-borne illness and Groupers are commonly reported as a source of CFP due to their position in the food chain. With the role of recent climate change on harmful algal blooms, CFP cases might become more frequent and more geographically widespread. Since there is no appropriate treatment for CFP, the most efficient solution is to regulate fish consumption. Such a strategy can only work if the fish sold are correctly identified, and it has been repeatedly shown that misidentifications and species substitutions occur in fish markets.

**Methods:**

We provide here both a DNA-barcoding reference for groupers, and a new phylogenetic reconstruction based on five genes and a comprehensive taxonomical sampling. We analyse the correlation between geographic range of species and their susceptibility to ciguatera accumulation, and the co-occurrence of ciguatoxins in closely related species, using both character mapping and statistical methods.

**Results:**

Misidentifications were encountered in public databases, precluding accurate species identifications. Epinephelinae now includes only twelve genera (vs. 15 previously). Comparisons with the ciguatera incidences show that in some genera most species are ciguateric, but statistical tests display only a moderate correlation with the phylogeny. Atlantic species were rarely contaminated, with ciguatera occurrences being restricted to the South Pacific.

**Conclusions:**

The recent changes in classification based on the reanalyses of the relationships within Epinephelidae have an impact on the interpretation of the ciguatera distribution in the genera. In this context and to improve the monitoring of fish trade and safety, we need to obtain extensive data on contamination at the species level. Accurate species identifications through DNA barcoding are thus an essential tool in controlling CFP since meal remnants in CFP cases can be easily identified with molecular tools.

## Introduction

Large carnivorous fishes associated with coral reefs are frequently contaminated by toxins responsible for ciguatera fish poisoning (CFP) in tropical and subtropical waters [1], [2]. CFP is a food-borne disease contracted by the consumption of finfish that have accumulated lipid-soluble toxins produced by microalgae (dinoflagellates) of the genus *Gambierdiscus* in their flesh and viscera. Dinoflagellates produce gambiertoxins which are first accumulated in the viscera of herbivorous fish and are further accumulated and converted to ciguatoxins in the flesh of larger carnivorous species. For the purposes of this report, we define ciguateric as possessing the ability to accumulate ciguatoxins and cause ciguatera fish poisoning. At least three groups of ciguatoxins have been identified: Pacific (P-CTX), Indian Ocean (I-CTX) and Caribbean (C-CTX) [3], [4]. While the gambiertoxin precursors for P-CTX have been identified, the corresponding precursors for I-CTX and C-CTX have yet to be identified, let alone a thorough examination of which dinoflagellates produce them. This disease produces several gastrointestinal, neurological and cardiac symptoms a few minutes to a few hours after ingestion of contaminated seafood [5]. Although there are reports of symptom amelioration with some interventions (e.g. IV mannitol), no efficient treatment exists so far [6]. It is a significant public health problem, especially in the South Pacific but also in the United States, where it is responsible for one of the highest reported incidence of seafood-borne illness [1]. Although CFP was historically restricted to tropical and sub-tropical regions, case reports are increasingly seen in higher latitudes with escalating global trade and movement of seafood products [1], [7]. The incidence of ciguatera, as well as the species of fish that are potentially poisonous, vary from region to region [2]. Precise information about the distribution of ciguatera-carrying species can be obtained from epidemiological data collected by research and health organisations in each country or region, but this depends heavily on correct species identification and is highly dependent on the intensity of data collection. An additional problem pointed out by several authors is the role of recent climate change on harmful algal blooms (HAB), including *Gambierdiscus* ssp. [1], [8], [9]. The abundance of *G*. spp. correlates positively with elevated sea surface temperature [8]. CFP cases might therefore become more frequent and more geographically widespread as an indirect consequence of climate change (review in [1]). Moreover, coral reefs perturbations, such as hurricanes or bleaching events, also free up space for microalgae to colonize. Even human activities altering the environment such as petroleum production platform building can contribute to the HAB [10]. Therefore, populations from developing countries, already facing these disturbances, appeared to be particularly exposed to the intensification of CFP.

Since there is no appropriate treatment for CFP (for a review, see [6]), the most efficient solution is to regulate fish consumption [11], [12]. Lewis [13], and more recently Clua et al. [11] recommended banning some specific species and sizes from fish markets. However, such a strategy can only work if the fish sold are correctly identified and labelled, and it has been repeatedly shown that misidentifications and species substitutions commonly occur in fish markets [14]–[16].

Groupers (Epinephelidae: rockcods, coralgroupers, hinds, and lyretails) are one of the families most commonly reported as a source of ciguatera poisoning [11]. Some grouper species, like *Plectropomus laevis* and *Cephalopholis argus*, are known to be especially contaminated by ciguatera toxins [17]–[19]. Large individuals are generally more toxic than small ones since ciguatoxins accumulate in fish via the food chain [11], [20]. For instance, specimens belonging to the potentially ciguatoxic fish species *Epinephelus fuscoguttatus* and *Variola louti* are considered dangerous only if they weigh more than 13 and 1.7 kg respectively [20]. Because they are widely distributed in warm and temperate shore waters, from surface to deep-sea, and adults of some species reach 3 m in length and 400 kg [18], groupers represent a considerable economic value in tropical and subtropical regions and most particularly in south-east Asia [21]–[24]. They are a major component of the artisanal fisheries resource especially in the south Pacific [18]. Global capture fisheries production has increased from approximately 214,000 tons in 1999 to more than 275,000 tons in 2009 [25]. Grouper aquaculture was first introduced in the early 1970s and is now widely practised throughout Southeast Asia [21]. Global grouper aquaculture production has increased tremendously due to increasing demand, from 60,000 tons in 1990 to 200,000 tons in 2007. The premium price of groupers can reach US$ 100/kg in the Chinese live fish markets [21].

Although groupers are large fish and supposedly easily identifiable, comprehensive and reliable species identification tools are rare and a good taxonomic framework is also necessary. Even when intact adult specimens are available (which is generally not the case for food-borne poisoning cases) the morphological characters used to discriminate species can be subtle, making identification difficult even for trained taxonomists. Moreover, accessing the historical literature and assessing the validity of species with a controversial taxonomic history are challenging tasks, even for experts [26].

Some rapid and reliable species identification tools such as DNA barcoding have been developed to facilitate species identification [26]–[30]. Given the estimated $US200 billion annual value of fisheries worldwide, the Fish Barcode of Life campaign (FISH-BOL) initiative, as a part of the International Barcode of Life Project (iBOL; http://www.ibolproject.org), is addressing socially relevant questions concerning market substitution and quota management of commercial fisheries (http://www.fishbol.org), with a special focus on developing countries [31]. However, species identification tools require complete and reliable databases. Indeed, DNA databases play a key role for the species identification of groupers, and more generally, for seafood, as non-specialists use essentially those databases to identify species for which they often have access to tissue samples only. The Epinephelidae comprise about 163 species [32] among which 106 are recorded in BOLD (942 public sequences in February 2014). The incompleteness of the reference datasets is a well-identified problem for species identification [33], which can be slightly alleviated if the marker used for identification is also relevant for phylogeny. In such a case, and if the taxonomic framework is accurate, species not represented in the database might still be assigned to clades or higher rank groups, like genera. Completing the largest molecular identification dataset (the cytochrome oxidase 1 of the Barcode of Life project), combined with an accurate study of the relationships of groupers, will help the management of grouper diversity through easier and more accurate identifications. Much remains to be done on both of these aspects, as the phylogenetic framework of the group has undergone many changes recently, and is yet incomplete.

The relationships of the Epinephelidae, recently raised to family rank by Smith and Craig [34], are indeed not yet totally resolved. Epinephelidae were previously a subfamily (Epinephelinae) included with Serraninae and Anthiinae among Serranidae [18], [35]. The relationships within the former Serranidae, as well as the composition of the family, have been the object of much discussion. Two molecular studies including the Serranidae showed the non-monophyly of the family [34], [36]. Smith and Craig [34] grouped Serraninae and Anthiinae in the Serranidae and raised the subfamily Epinephelinae to the family rank Epinephelidae. On the other hand, Lautrédou et al. [36] showed the polyphyly of Serranidae (with the Serraninae – Anthiinae composition), while recovering an Anthiinae and Epinephelidae clade. Craig et al. [32] defined four subfamilies in Epinephelidae: Diploprioninae, Epinephelinae, Grammistinae and Liopropominae, corresponding to the four previous tribes Diploprionini, Epinephelini, Grammistini, Liopropomini. In their molecular phylogeny, Craig and Hastings [37] attempted to resolve the phylogeny of the Epinephelidae using an almost complete species sampling within the genus *Epinephelus*, and several specimens of other subfamilies, using two mitochondrial and two nuclear markers. They proposed taxonomic changes for species of the subfamily Epinephelinae to reflect their phylogenetic position. For instance, they included *Cromileptes altivelis* and *Anyperodon leucogrammicus* in *Epinephelus* and they moved *Epinephelus septemfasciatus* and *E. ergastularius* to *Hyporthodus*. However, many nodes of their phylogeny lacked robustness. Because of the absence of morphological differences between the genera *Anyperodon*, *Cromileptes* and *Epinephelus*, Craig et al. [32] retained the monotypic genera *Anyperodon* and *Cromileptes*.

To further the study of the relationships between the genera, we sequenced five markers, two mitochondrial, Cytochrome Oxidase Subunit I (COI) and 16S ribosomal RNA (16S) and three nuclear, Rhodopsin (Rh), Titin-like protein (TMO-4C4) and Polycystic kidney disease 1 protein (Pkd1). We choose to include the reference barcoding marker COI to provide a simple and reliable tool for species identification to the non-specialist community (fisheries, governmental organisations, etc.). As ciguatera occurrence has not yet been studied with regard to the evolutionary relationships of the ciguateric fish replaced in their evolutionary context, the second aim of this paper is to map the high risk species for ciguatera fish poisoning into the phylogeny using published information about ciguatera-prone species. We statistically test whether high risk species are closely related and could therefore have inherited their susceptibility to ciguatera from their common ancestor.

## Methods

### Taxonomic sampling

#### Sequencing fresh material collection

Fishes collected from different localities (Table 1) in 2009–2011 were dead at the time we acquired them for study, having been commercially caught, and available for purchase at the Nouméa fish market. Each individual was morphologically identified according to Heemstra & Randall [18], measured, weighed, and photographed, and a tissue sample was collected and preserved in absolute ethanol until DNA extraction. Several specimens per species were sequenced to evaluate intraspecific variation and to corroborate identification (data not shown). Additional fish tissues were obtained from colleagues (see acknowledgements) or bought at fish markets, and also preserved in absolute ethanol. For these tissues, no photograph was available; the identification of these tissues was checked by a BLAST search in BOLD [38] followed by a thorough evaluation of the results. Samples and results not corresponding to a higher sequence and identification quality standard were discarded.

**Table 1:** List of fish species, specimen vouchers localities and sequence accession numbers.

Family	Group: subfamily /tribe	Species	Accession Number					Specimen	Locality
			Mitochondrial		Nuclear			Voucher	
			COI	16S	TMO-4C4	Rhodopsin	Pkd1		
Epinephelidae	Diploprioninae	*Belonoperca chabanaudi*	JQ431484_v_*	JX094024_v_*	JX093971_v_*			MNHN 2008-1159	Moorea, French Polynesia
		*Diploprion bifasciatum*	KM077912_v_*	KM077970_v_*	KM078001_v_*			MNHN-icti-2815	Queensland, Australia
	Liopropominae	*Liopropoma fasciatum*	JX093903*	JX093999*	JX093972*	JX093952*	JX093928*		Ecuador
		*Liopropoma lunulatum*	JQ431888_v_	JX094023_v_*	JX093974_v_*	JX093953_v_*	JX093929_v_*	MNHN 2008-1023	Moorea, French Polynesia
		*Liopropoma pallidum*	JQ431890_v_	JX094020_v_*	JX093973_v_*			MNHN 2008-0793	Moorea, French Polynesia
	Grammistinae	*Aporops bilinearis*	JQ431457_v_	JX094016_v_*	AY949271			MNHN 2008-0307	Moorea, French Polynesia
		*Grammistes sexlineatus*	JQ431776_v_	AY539050	AY539458			MNHN 2008-1105	Moorea, French Polynesia
		*Grammistops ocellatus*	JQ431778_v_	JX094021_v_*	X093975_v_*			USNM 391102	Moorea, French Polynesia
		*Pogonoperca punctata*	JX093904*	JX093998*	JX093976*	JX093951*	JX093927*		
		*Pseudogramma gregoryi*	GU225013	AY947571	AY949213				
		*Pseudogramma polyacantha*	JQ432063_v_	JX094018_v_*	JX093977_v_*			MBIO509.4	Moorea, French Polynesia
		*Rypticus saponaceus*	JX093905_v_*	JX094000_v_*	JX093978_v_*	JX093956_v_*	JX093932_v_*	MNHN2002-158	Ghana
		*Suttonia lineata*	JQ432178						
	Epinephelinae	*Cephalopholis argus*	JQ431565_v_	JX094015_v_*	JX093979_v_*	JX093958_v_*	JX093934_v_*	MNHN 2008-0229	Moorea, French Polynesia
		*Cephalopholis boenak*	KM077907_v_*	KM077965_v_*	KM077996_v_*	KM077936_v_*	KM077879_v_*	MNHN-icti-2875	New Caledonia
		*Cephalopholis colonus*	GU440449						
		*Cephalopholis cruentata*	GU225172	AF297323	AY949266				
		*Cephalopholis cyanostigma*	KM077908_v_*	KM077966_v_*	KM077997_v_*			MNHN-icti-2821	Queensland, Australia
		*Cephalopholis formosa*	FJ583004	AY947603	EF517741				
		*Cephalopholis fulva*	FJ583007	AF297292	AY949282				
		*Cephalopholis hemistiktos*	HQ149822						
		*Cephalopholis igarashiensis*	EU871685	AY947599	AY949292				
		*Cephalopholis leopardus*	FJ583010	AY947560	AY949323				
		*Cephalopholis microprion*	FJ237608						
		*Cephalopholis miniata*	KM077909_v_*	KM077967_v_*	KM077998_v_*	KM077937_v_*	KM077880_v_*	MNHN-icti-3008	New Caledonia
		*Cephalopholis rogaa*	JQ349677	EF503626	EF517737				
		*Cephalopholis sexmaculata*	JQ431572_v_	JX094019_v_*	JX093980_v_*	JX093959_v_*	JX093935_v_*	MNHN 2008-0754	Moorea, French Polynesia
		*Cephalopholis sonnerati*	JX093918*	JX094007*	JX093981*	JX093960*	JX093936*		New Caledonia
		*Cephalopholis spiloparaea*	KM077910_v_*	KM077968_v_*	KM077999_v_*	KM077938_v_*	KM077881_v_*	MNHN-icti-2961	New Caledonia
		*Cephalopholis urodeta*	KM077911_v_*	KM077969_v_*	KM078000_v_*	KM077939v*	KM077882_v_*	MNHN-icti-2886	New Caledonia
		*Dermatolepis dermatolepis*	JX093917*	JX094006*	JX093982*	JX093955*	JX093931*		Ecuador
		*Epinephelus adscensionis*	FJ583396	AY539049	AY949284				
		*Epinephelus aeneus*	KM077913_v_*	KM077971_v_*	KM078002_v_*	KM077940_v_*	KM077883_v_*	MNHN-icti-2842	Senegal^1^
		*Epinephelus akaara*	GBGC7768	DQ154107	EF517707				
		*Epinephelus altivelis*	JX093906*	JX094001*	JX093983*	JX093957*	JX093933*		Origin unknown, Aquarium
		*Epinephelus amblycephalus*	JX093910*	JX094009*	JX093984*	JX093961*	JX093937*		South China Sea^1^
		*Epinephelus analogus*	JX093915*	JX094003*	AY949220				Ecuador
		*Epinephelus areolatus*	KM077914_v_*	KM077972_v_*	KM078003_v_*	KM077941_v_*	KM077884_v_*	MNHN-icti-2996	New Caledonia
		*Epinephelus awoara*	JX093913*	JX094013*	JX093985*	JX093969*	JX093945*		South China Sea^1^
		*Epinephelus bleekeri*	JX093911*	JX094011*	JX093986*	JX093962*	JX093938*		South China Sea^1^
		*Epinephelus bruneus*	JX093909*	JX094010*	JX093992*	JX093968*	JX093944*		South China Sea^1^
		*Epinephelus chlorostigma*	JQ412501_v_	KM077973_v_*	KM078004_v_*	KM077942_v_*	KM077885_v_*	MNHN-icti-2145	New Caledonia
		*Epinephelus clippertonensis*	JX093914*	JX094002*	JX093987*	JX093964*	JX093940*		Clipperton Island
		*Epinephelus coeruleopunctatus*	KM077915_v_*	KM077974_v_*	KM078005_v_*	KM077943_v_*	KM077886_v_*	MNHN-2006-1706	New Caledonia
		*Epinephelus coioides*	KM077916_v_*	KM077975_v_*	KM078006v*	KM077944_v_*	KM077887_v_*	MNHN-icti-2994	New Caledonia
		*Epinephelus corallicola*	JX093908*	JX094008*	JX093988*	JX093967*	JX093943*		South China Sea^1^
		*Epinephelus cyanopodus*	JQ412502_v_	KM077976_v_*	KM078007_v_*	KM077945_v_*	KM077888_v_*	MNHN-icti-2148	New Caledonia
		*Epinephelus diacanthus*	EF609517	AY947619	AY949274				
		*Epinephelus fasciatomaculosus*	EF607565	AY947622	EF517717				
		*Epinephelus fasciatus*	JX093907*	JX094005*	JX093990*	JX093970*	JX093946*		Gulf of Aqaba
		*Epinephelus flavocaeruleus*	SAIAB330-06_b_	AY947607	EF517731				
		*Epinephelus fuscoguttatus*	EU600139	JF750752	EF517713				
		*Epinephelus hexagonatus*	JQ431719_v_	JX094017_v_*	JX093991_v_*	JX093966_v_*	JX093942_v_*	MNHN 2008-0338	Moorea, French Polynesia
		*Epinephelus howlandi*	KM077917_v_*	KM077977_v_*	KM078008_v_*	KM077946_v_*	KM077889_v_*	MNHN-icti-2981	New Caledonia
		*Epinephelus lanceolatus*	NC_011715	AY947588	EF517736				
		*Epinephelus latifasciatus*	EU014219	DQ088044	EF517724				
		*Epinephelus leucogrammicus*	KM077918_v_*	KM077978_v_*	KM078009_v_*	KM077947_v_*	KM077890_v_*	MNHN-icti-2993	New Caledonia
		*Epinephelus longispinis*	EF609522	EF213704	EF517706				
		*Epinephelus macrospilos*	SAIAB547-07_b_	AY731072	AY949238				
		*Epinephelus maculatus*	KM077919_v_*	KM077979_v_*	KM078010_v_*	KM077948_v_*	KM077891_v_*	MNHN-icti-2907	New Caledonia
		*Epinephelus malabaricus*	KM077920_v_*	KM077980_v_*	KM078011_v_*	KM077949_v_*	KM077892_v_*	MNHN-icti-3018	New Caledonia
		*Epinephelus melanostigma*	JQ349966	EF503624	EF517730				
		*Epinephelus merra*	KM077921_v_*	KM077981_v_*	KM078012_v_*	KM077950_v_*	KM077893_v_*	MNHN-icti-2963	New Caledonia
		*Epinephelus multinotatus*	SAIAB149-06_b_						
		*Epinephelus ongus*	KM077922_v_*	KM077982_v_*	KM078013_v_*	KM077951_v_*	KM077894_v_*	MNHN-icti-3030	New Caledonia
		*Epinephelus polyphekadion*	KM077923_v_*	KM077983_v_*	KM078014_v_*		KM077895_v_*	MNHN-icti-2979	New Caledonia
		*Epinephelus polyphekadion*				KM077952_v_*		MNHN-icti-3015	New Caledonia
		*Epinephelus quoyanus*	DQ107861	JX094014_v_*	JX093993_v_*			MNHN-icti-2819	Queensland, Australia
		*Epinephelus retouti*	KM077924_v_*	KM077984_v_*	KM078015_v_*	KM077954_v_*	KM077896_v_*	MNHN-icti-2967	New Caledonia
		*Epinephelus rivulatus*	KM077925_v_*	KM077985_v_*	KM078016_v_*	KM077955_v_*	KM077897_v_*	MNHN-icti-2890	New Caledonia
		*Epinephelus sexfasciatus*	EF607564	DQ067310	EF517716				
		*Epinephelus spilotoceps*	KM077926_v_*	KM077986_v_*	KM078017_v_*	KM077956_v_*	KM077898_v_*	MNHN-icti-3050	Maldives^1^
		*Epinephelus tauvina*	JX093916*	JX094004*	JX093994*	JX093965*	JX093941*		Gulf of Aqaba
		*Epinephelus undulosus*	EF609352	DQ088041	EF517704				
		*Hyporthodus haifensis*	CSFOM034-10_b_						
		*Hyporthodus septemfasciatus*	DQ107851	AY947559	AY949247				
		*Mycteroperca bonaci*	GU225646	DQ267150	AY949270				
		*Mycteroperca canina*	CSFOM032-10_b_	AY947585	AY949294				
		*Mycteroperca costae*	KM077928_v_*	KM077988_v_*	KM078019_v_*	KM077957_v_*		MNHN-icti-2868	Tunisia^1^
		*Mycteroperca costae*					KM077899_v_*	MNHN-icti-2859	Tunisia^1^
		*Mycteroperca interstitialis*	FJ583668	AY947632	AY949221				
		*Mycteroperca jordani*	GU440412	AF297329	AY949303				
		*Mycteroperca marginata*	KM077929_v_*	KM077989_v_*	KM078020_v_*	KM077958_v_*	KM077900_v_*	MNHN-icti-2854	Senegal
		*Mycteroperca microlepis*	JN021310	AF297312	AY949253				
		*Mycteroperca morrhua*	KM077930_v_*	KM077990_v_*	KM078021_v_*	KM077959_v_*	KM077901_v_*	MNHN-icti-2947	New Caledonia
		*Mycteroperca poecilonota*	SAIAB576-07_b_						
		*Mycteroperca rubra*	CSFOM051-10_b_	AY947587	AY949255				
		*Mycteroperca xenarcha*	GU440413						
		*Plectropomus areolatus*	JN242591	EF213706	EF517750				
		*Plectropomus laevis*	KM077932_v_*	KM077992_v_*	KM078023_v_*	KM077961_v_*	KM077903_v_*	MNHN-icti-3012	New Caledonia
		*Plectropomus leopardus*	KM077933_v_*	KM077993_v_*	KM078024_v_*	KM077962_v_*	KM077904_v_*	MNHN-icti-2883	New Caledonia
		*Plectropomus maculatus*	DQ107911	JF750755	EF517751				
		*Saloptia powelli*	JQ432090_v_	JX094022_v_*	JX093995_v_*	JX093954_v_*	JX093930_v_*	MNHN 2008-1014	Moorea, French Polynesia
		*Triso dermopterus*	DQ107934						
		*Variola albimarginata*	KM077934_v_*	KM077994_v_*	KM078025_v_*	KM077963_v_*	KM077905_v_*	MNHN-icti-2999	New Caledonia
		*Variola louti*	KM077935_v_*	KM077995v*	KM078026_v_*	KM077964_v_*	KM077906_v_*	MNHN-icti-2964	New Caledonia
Anthiinae		*Pseudanthias hypselosoma*	JX093919*	JX094027*	JX093996*	JX093950*	JX093926*		New Caledonia^1^
		*Pseudanthias pleurotaenia*	JX093920*	JX094026*	AY949308*	JX093949*	JX093925*		
		*Pseudanthias tuka*	JX093921*	JX094025*	JX093997*	JX093948*	JX093924*		
Cirrhitidae		*Cirrhitus pinnulatus*	JQ431641			KC222240_b_	JX628396		
		*Cirrhitus rivulatus*		AY539059	AY539467				
Harpigiferidae		*Harpagifer kerguelenensis*	EATF605_b_	AY539063	AY539471	EATF605_b_	JQ688766		
Niphonidae		*Niphon spinosus*	EF143386	AY947575	AY949210				
Percidae		*Perca fluviatilis*	AP040-12_b_	GU018097		AP040-12_b_	JX628360		
		*Perca flavescens*			AY539463				
Scorpaenidae	Congiopodidae	*Zanclorhynchus spinifer*	AP139-12_b_	AY538999	AY539413	EU638021	JX628429		
	Cyclopteridae	*Cyclopterus lumpus*	AP041-12_b_	AY539043	AY539451	AP041-12_b_	JX628359		
	Scorpaeninae	*Pontinus* longispinis		AY538982	AY539398				
		*Pontinus macrocephalus*	JX093922*			JX093947*	JX093923*		Philippines
		*Scorpaenopsis macrochir*		AY538987	AY539402				
		*Scorpaenopsis possi*	JQ432137			KC222244_b_	JX628403		
	Sebastinae	*Helicolenus dactylopterus*	AP121-12_b_	AY538975	AY539391	AP121-12_b_	JX628383		
		*Trachyscorpia cristulata*	AP111-12_b_	AY538980	AY539396	AP111-12_b_	JX628374		
	Synanceinae	*Synanceia verrucosa*	JQ432179	AY538995	AY539410	KC222242_b_	JX628405		
Serraninae		*Centropistis striata*	HQ024935	AY072667	AY949216				
		*Serranus tigrinus*	FJ584106	AY072688	AY949259				
Trachinidae		*Trachinus draco*	AP104-12_b_	AY539068	AY539476	AP104-12_b_	JX628367		

(^1^) Fish specimen collected at Nouméa fish market or obtained through colleagues, (*) new sequences, (_b_) BOLD accession number, (_v_) sequence corresponds to voucher indicated in Table.

#### Publicly available sequences

All available COI, 16S and TMO-4C4 sequences of Epinephelidae and some outgroup sequences were downloaded from public sequence databases (Barcode of life Database, GenBank Nucleotide). All sequences were controlled for contamination, indels, and stop-codons indicating possible pseudogenes [39]. We followed the classification of Craig and Hastings [37]. When necessary, we amended the species name to agree with the genus gender (*Cephalopholis rogaa*, *Mycteroperca canina* and *M. marginata*) as per the recommendations of the International Code of Zoological Nomenclature (Fourth Edition).

Problems were identified within both GenBank and BOLD sequences, and some sequences available in those databases were therefore not integrated in our dataset. Database sequences presented either: (i) taxonomic problems, such as high genetic divergence within species or erroneous species identification and (ii) nomenclatural problems like the use of invalid species names.

The specimen identification for some sequences was problematic. While there were often several specimens attributed to a species in the databases, high intraspecific variability for COI between specimens was observed for some of them. For instance, the species *E. macrospilo*s and *E. tauvina* have a COI genetic divergence within species of 7.4 and 6.2% respectively, values very largely above what is known of fish intraspecific diversity for this marker [40]. These species, and some others, were represented by multiple, disjoint clusters. For example, for *Epinephelus tauvina* one cluster of database sequences included our seven *E. tauvina* sequences, while another cluster matched with our eleven sequences of *E. coioides*. The species *E. akaara*, *E. amblycephalus, E. diacanthus*, *E. longispinis*, *E. macrospilos*, *E. sexfasciatus* and *Variola louti* were especially subject to such high intraspecific divergence, a well-known indication of misidentification or unresolved taxonomic issues. These reliability problems are all the more important when the molecular identification has medical applications, such as determining whether a sample can come from a ciguateric species. In order to select the more reliably identified specimens, we checked the supplementary information (voucher, geographic information) to corroborate the identification of the specimens. Moreover, in case of relatively high COI intraspecific variability within species (>1%), we selected the specimen collected closest to the type-locality to minimize taxonomic misidentifications and errors linked to possible cryptic species.

Second, the databases use some invalid names. While spelling errors are relatively straightforward to identify, like the use of *Cephalopholis miniatus* instead of *C. miniata* or *Pseudogramma polyacanthum* instead of *P. polyacantha*, nomenclaturally invalid names were also present in the databases. For instance, *E. ‘fario’* (Thunberg, 1793), represented by eight specimens in GenBank including two COI sequences, has been pointed out by Heemstra and Randall [18] as a synonym of *E. longispinis* (Kner, 1864) and as a *nomen dubium*. Randall and Heemstra [41] also regarded this species as unidentifiable. We did not include these sequences in our dataset.

### Ethics statement

Fish were dead at the time we acquired them for study, having been commercially caught, and available for purchase at the Nouméa fish market; no permits were required for the described study, which complied with all relevant regulations.

### DNA extraction, amplification, and sequencing

DNA was extracted from tissue samples using NucleoSpin 96 tissue kit (Macherey-Nagel, Düren, Germany) and five genes were amplified (Table 2).

**Table 2:** List of the primers used in this study; T° of hyb: temperature of hybridisation used to amplify the marker.

Gene	Fragment size	Name	Primers	T° of hyb	Sources
COI	≈650 bp	FishF1	5′-TCAACCAACCACAAAGACATTGGCAC-3′	48°C	[30]
		FishR1	5′-TAGACTTCTGGGTGGCCAAAGAATCA3′		[30]
16S	≈410 bp	16SarL	5'-CGCCTGTTTATCAAAAACAT-3'	54°C	[75]
		16SbrH	5'-CCGGTCTGAACTCAGATCACGT-3'		[75]
Rhodo	≈720 bp	Rh193	5'-CNTATGAATAYCCTCAGTACTACC-3'	52°C	[76]
		Rh1039r	5'-TGCTTGTTCATGCAGATGTAGA-3'		[76]
Pkd1	≈850 bp	Pkd1F62	5'-CATGAGYGTCTACAGCATCCT-3'	50°C	[77]
		Pkd1R952	5'-YCCTCTNCCAAAGTCCCACT-3'		[77]
TMO-4C4	≈540 bp	TMOF1	5′-CCTCCGGCCTTCCTAAAACCTCTC-3′	55°C	[78]
		TMOR1	5'-CATCGTGCTCCTGGGTGACAAAGT-3′		[78]

Each PCR reaction was performed in a 20 µl final volume, containing 2 ng of DNA, 1X reaction buffer, 0.26 mM dNTP, 0.8 µM of each primer, 5% DMSO and 1.5 units of Taq polymerase (Qiagen). Thermocycles consisted of an initial denaturation step at 94°C for 2′, followed by 37–55 cycles of denaturation at 94°C for 30″, annealing at 48–55°C for 40″ (Table 2) and extension at 72°C for 1′. The final extension was conducted at 72°C for 10′. Purification and cycle-sequencing reactions were performed at the Génoscope (Évry, France), using the BigDye Terminator version 3 sequencing kit, the GeneAmp PCR System 9700 and a capillary ABI3730 DNA Analyser, all from Applied Biosystems. Sequences were edited and assembled using Sequencher 4.9 (Gene Codes Corporation, Ann Arbor, MI, USA). Sequences for this study were deposited in GenBank (Table 1).

### Phylogenetic analyses and mapping

All markers were sequenced for both directions to confirm accuracy for each individual specimen. However, a few sequences could not be obtained (Table 1). Sequences were aligned using ClustalW as implemented in BioEdit version 7.0.5.3 [42] or by eye. The accuracy of automatic alignments was assessed by eye. 16S sequences were aligned manually and two portions (between positions 240–300 and 347–450) were removed from the alignment due to hypervariable regions that could not be aligned reliably.

94 species of Epinephelidae were included in our analyses, 2 species for Diploprioninae, 81 species for Epinephelinae, 8 species for Grammistinae and 3 species for Liopropominae.

To study (i) the monophyly of the Epinephelidae (*i.e* according to traditional classification Epinephelinae minus *Niphon*) and (ii) the relationships within this family, outgroups were chosen in the sub-families of Serranidae, the Serraninae and the Anthiinae [34]. We also included multiple, non-monophyletic outgroups: specimens from Perciformes (Cirrhitidae, Harpagiferidae, Niphonidae, Percidae, Trachinidae) and from Scorpaeniformes (Congiopodidae, Cyclopteridae, Scorpaeninae, Sebastinae, Synanceinae) placed as close relatives of Epinephelidae in the study of Lautrédou et al. [36].

### Phylogenetic analyses of the Epinephelidae

All markers were first analysed separately, tested for incongruence and, since none was detected, concatenated in two different datasets. The combined and separate analyses have three different aims (i) obtaining the most robust phylogenetic reconstruction while including a maximal species representation, *i.e.* including downloaded sequences (dataset 1), (ii) maximising the number of markers, even if it includes less species (dataset 2), and (iii) best representing the diversity of the Epinephelidae with the COI gene alone (dataset 3). Dataset 1 included the concatenated COI, 16S and TMO-4C4 sequences. Dataset 2 included all five concatenated markers, *i.e.* COI, 16S, TMO-4C4, Rhodopsin and Pkd1 sequences.

The best-fitting models of nucleotide evolution for each gene and for the concatenation were determined based on the Akaike Information Criterion (AIC) implemented in ModelTest 2.3 [43] in conjunction with PAUP 4.0b10 [44]. The GTR + I + Γ model was selected for each marker and for the two datasets.

Trees were inferred using two probabilistic approaches: maximum likelihood with a non-parametric bootstrap (BP) using RAxML 7.2.8 [45], [46] and Bayesian Inference [47]. Maximum likelihood (ML) analyses were carried out online on the CIPRES Science Gateway (The CIPRES Portals. URL: http://www.phylo.org/sub_sections/portal) with RAxML-HPC BlackBox (7.2.7) [45]. Datasets were partitioned by codon position for each marker (except 16S) and by marker and by codon position for datasets 2 and 3. BI analyses were performed with MRBAYES version 3.2.1 [47] using 75,000,000, 75,000,000, and 30,000,000 generations for datasets 1, 2 and 3 respectively, with sampling every 1,000 generations and four Metropolis-coupled Markov chains Monte Carlo (MCMCMC). The parameter estimates and convergence were checked using Tracer version 1.4 [48]. The first 25% of sampled trees were considered burn-in trees, and discarded prior to constructing a 50% majority rule consensus trees. Posterior probabilities (PP) (Bayesian analysis) and Bootstrap probabilities (BP) were used as indicators of node credibility; P95% was considered significant [49]. Two independent analyses were conducted to check for convergence of the results.

### Mapping of potential ciguatera poisonous species

Potentially ciguatera affected species were mapped onto the phylogenetic tree inferred from all the available reliable sequences for the COI fragment. Although several relationships were not resolved compared to the combined analyses, the COI topology is congruent with the combined analyses topologies, and moreover best represents the diversity of the Epinephelidae.

A large number of studies on ciguatera were reviewed to establish a list of ciguatera affected species [7], [11], [12], [19], [20], [50]–[59]. However, there were very few publications where species names were precisely indicated. We ended up by using five [11], [20], [53], [54], [58], as well as the available ciguatera data in Fishbase database [17]. To detect a potential pattern in the evolution of the occurrence of ciguatera in Epinephelinae, we quantified the strength of phylogeny-trait association. The MrBayes phylogeny from the dataset 3 was used as an input in the BaTS software [60], with the occurrence of the ciguatera coded as absent/present. 10.000 trees from the posterior set of trees from MrBayes analysis were randomly selected after removing the first 7.5 million generations as a burnin according to Tracer [48], and re-rooted using the outgroups and a custom-made R script [61] (script available on request). BaTS estimates several statistics: the parsimony score (PS), the association index (AI) and the monophyletic clade (MC) and tests their significance against a null distribution (obtained by reshuffling 100 times the ciguatera states on the tips). A strong phylogeny-trait association is identified by low PS and AI scores and a high MC score.

### Evolution of the ciguatera fish poisoning (CFP)

Ancestral character state reconstructions for ciguatera fish poisoning were conducted using the maximum-likelihood method implemented in Mesquite 2.75 [62]. Recognized species were assigned different states (following a bibliographic survey, see above): absence (0) and presence (1) of CFP. Ancestral states were reconstructed for all Bayesian trees retained from the analysis of the combined data set and their mean likelihood was then plotted on the maximum clade credibility tree.

## Results

### Phylogenetic relationships within Epinephelidae

Dataset length and number of variable sites are reported in Table 3. 47 and 58 sequences were obtained for the mitochondrial markers COI and 16S, respectively. 54 sequences were obtained for TMO-4C4, 50 for Rhodopsin and 50 for Pkd1.

**Table 3:** Dataset composition, marker size and information.

Dataset	Number of species	Markers	Length (bp)	Conserved sites	Variable sites
1	102	COI	651	372	279
		16S	410	254	156
		TMO-4C4	511	271	240
2	60	COI	651	382	269
		16S	410	278	132
		TMO-4C4	536	330	206
		Pkd1	861	422	439
		Rhodopsin	723	470	253
3	112	COI	651	371	280

**Figure 1:**
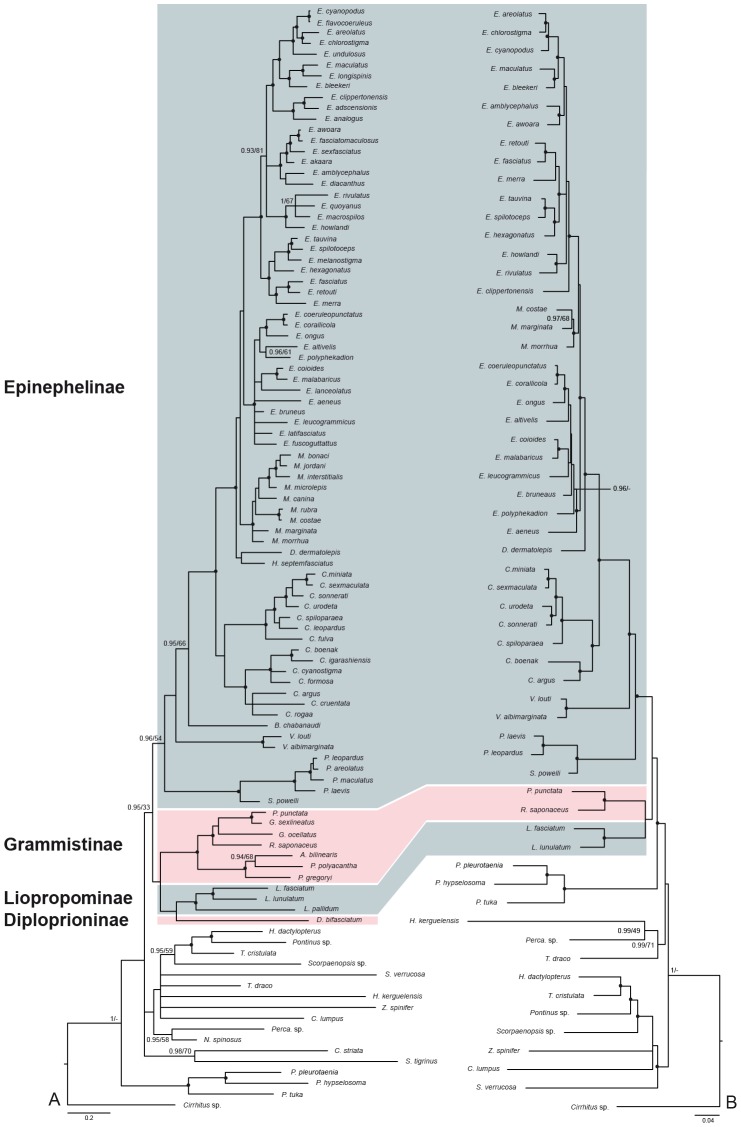
Phylogenetic relationships within the Epinephelidae. Bayesian inference phylogram obtained from phylogenetic analyses of the dataset 1 (A) based on the concatenation of three genes, COI, 16S and TMO-4C4, and the dataset 2 (B) obtained with the concatenation of five genes, COI, 16S, TMO-4C4, Rhodopsin and Pkd1, both under the GTR + I + Γ model. Epinephelidae are highlighted in colour (pink and blue). Each sub families are shown in alternate blue and pink colours. Values at nodes indicate Bayesian posterior probabilities (PP) and maximum likelihood bootstrap percentages (BP). Black circles indicate nodes supported by posterior probability ≥95% and ML bootstrap probability ≥75%.

The concatenated phylogeny (Fig. 1A) based on the three markers with the largest sampling (COI, 16S and TMO-4C4) recovered the monophyly of Epinephelidae comprising the four subfamilies, Diploprioninae, Epinephelinae, Grammistinae, and Liopropominae with a weak support. The Grammistinae was monophyletic. The three species of the genus *Liopropoma* of the Lioprominae were grouped. The Diploprioninae and the Epinephelinae were together not monophyletic because of the inclusion of *Belonoperca chabanaudi* within Epinephelinae. However, to the exception of the insertion of *B. chabanaudi*, all Epinephelinae specimens were grouped. In the Epinephelinae, the genera *Variola* and *Plectropomus* were together monophyletic with a strong support. *Saloptia* was sister-group to *Plectropomus*. *Cephalopholis* was monophyletic but divided in two robust groups with the inclusion of *C. colonus* and *C. rogaa* (earlier combinations: *Paranthias colonus* and *Aethaloperca rogaa*). *Epinephelus leucogrammicus* and *E. altivelis* (earlier combinations: *Anyperodon leucogrammicus* and *Cromileptes altivelis*, both monotypic genera) were included within the clade *Epinephelus*. *Mycteroperca* was sister-group to *Epinephelus* and monophyletic with a strong support. *D. dermatolepis* was sister-group to *Hyporthodus*, *Mycteroperca* and *Epinephelus*. The subfamily Grammistinae was sister-group to the Liopropominae and *Diploprion bifasciatum* from the Diploprioninae.

The concatenated phylogeny (Fig. 1B) based on all five markers (COI, 16S, TMO-4C4, Rhodopsin and Pkd1) confirmed the monophyly of Epinephelinae as well as the three subfamilies Epinephelinae, Grammistinae and Liopropominae. No specimen from the subfamily Diploprioninae was available for the Rhodopsin and Pkd1 markers; the monophyly of this subfamily cannot be evaluated with this second dataset.

Within Epinephelinae and as suggested by dataset 1, the monophyly of *Mycteroperca*, *Plectropomus* and *Variola* were well supported. *Cephalopholis* also constituted a robust monophylum with two clades, one containing *C. argus* and *C. boenak* and the other the rest of the species (as observed with the previous tree). *Epinephelus* was monophyletic with a weak support. *D. dermatolepis* was sister-group to a clade including *Epinephelus* and *Mycteroperca*.

Although several relationships were not resolved compared to the combined analyses (Fig. 1), the COI topology (Figure S1) is congruent with them, and has a much larger species sampling. The subfamilies Grammistinae and Liopropominae are monophyletic.

With the exception of the insertion of the species *Epinephelus poecilonotus* and *E*. *haifensis* included in the genera *Mycteroperca* and *Hyporthodus* respectively, all *Mycteroperca* and *Hyporthodus* species were grouped.

Like in the concatenated analyses (Figs. 1A-B), the monotypic genera *Anyperodon* and *Cromileptes* were together included in *Epinephelus*. The subfamily Diploprioninae including the three genera *Beloperca, Diploprion and Aulacocephalus* was not monophyletic, with the exclusion of *Beloperca chabanaudi*. *Diploprion bifasciatum* and *Aulacocephalus temminckii* constituted a robust clade.

### Mapping of potentially ciguatera affected species on the COI tree

**Figure 2:**
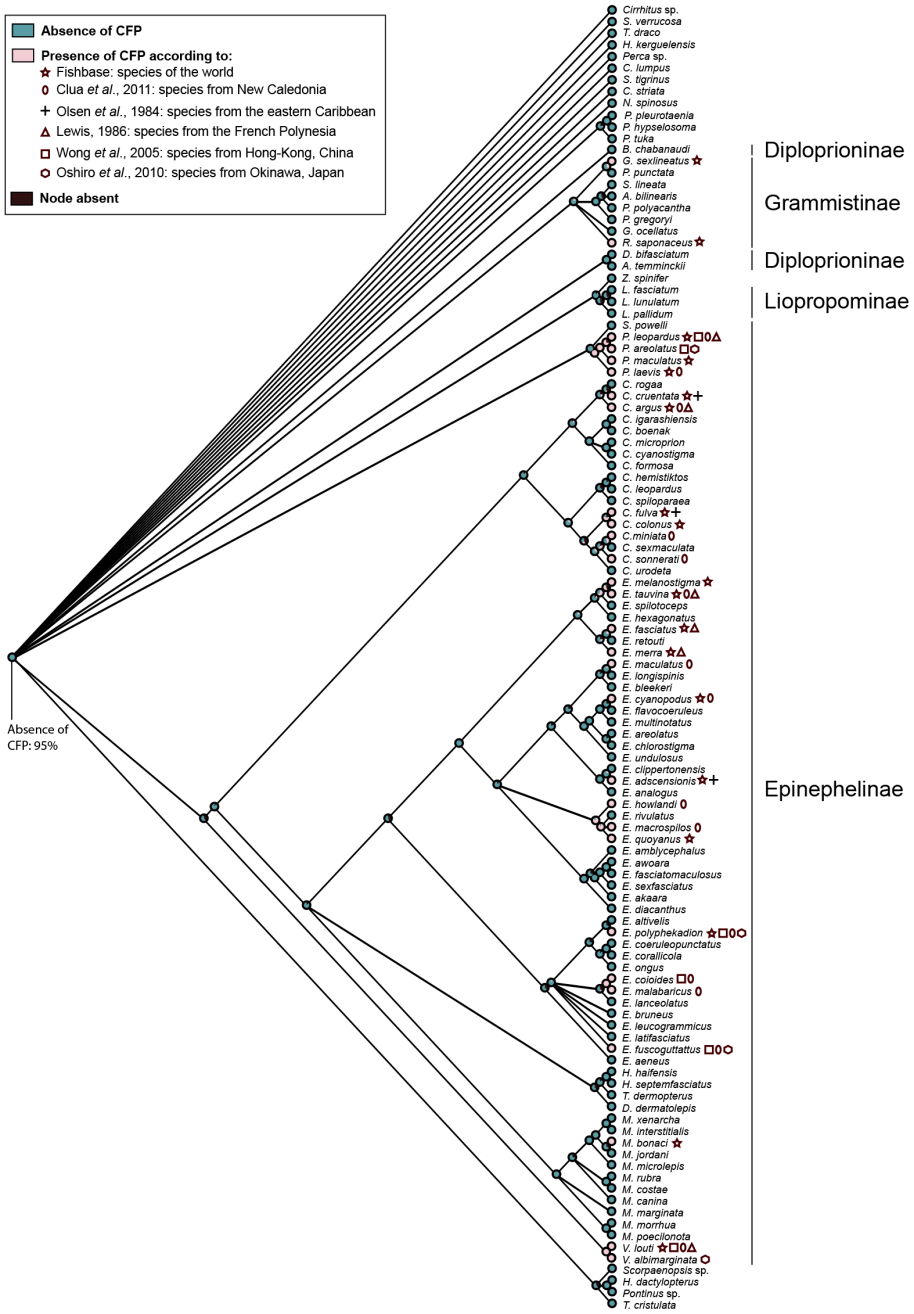
Ancestral ciguatera fish poisoning (CFP) reconstruction of Epinephelidae. Bayesian cladogram of the COI dataset with maximum likelihood estimates of ancestral CFP states. Pie charts correspond to average likelihoods for each state. Percentage values are given for nodes of interest.

Twenty nine ciguateric species were found in a review of the literature. Most of the species affected by ciguatera belong to the subfamily Epinephelinae (Fig. 2). Only two species, outside the Epinephelinae, *Grammistes sexlineatus* and *Rypticus saponaceus* are ciguateric. Within Epinephelinae, three genera contain multiple ciguateric species. All species included in the genera *Plectropomus* (4) and *Variola* (2) are ciguateric. In the genus *Epinephelus*, four species out of seven are ciguateric in one clade (*E. fasciatus*, *E. hexagonatus*, *E. melanostigma*, *E. merra*, *E. retouti*, *E. spilotoceps* and *E. tauvina*) (Fig. 2). In another, three species out of four are ciguateric (*E. quoyanus, E. macrospilos, E. howlandi and E. rivulatus*). The other ciguateric species are dispersed in clades where most species are not known to be affected. Within the other genera of Epinephelinae, *Mycteroperca* and *Hyporthodus*, only one species is ciguateric, *M. bonaci*.

The analysis of the presently available data on presence or absence of ciguatera fish poisoning (CFP) for each species mapped the absence of CFP as the ancestral state of Epinephelidae. The ancestral state analysis underlines the multiple appearances of CFP in this group with at least 10 events in the subfamily Epinephelinae (Fig. 2).

The Bayesian analysis of the phylogenetic signal (Table 4) revealed the occurrences of ciguatera in groupers species have a moderate non-random association with phylogeny; only the MC statistic for the occurrence of ciguatera shown significant P-value (p = 0.04), whereas the PS statistic shown a marginally significant P-value (p = 0.080). Both the AI statistic and the MC value for the ancestral state were not significant (p = 0.24 and p = 0.55, respectively).

**Table 4:** Results of the Bayesian phylogeny-trait association.

Statistic	Observed Distribution		Null Distribution		P-value
	observed mean	95% CI	null mean	95% CI	significance
AI	4.2	3.7–4.6	4.6	3.6–5.5	0.240
PS	23.7	23.0–24.0	26.3	23.3–28.7	0.080*
MC (absence of ciguatera)	6.6	6.0–9.0	6.1	4.1–7.9	0.550
MC (occurrence of ciguatera)	4.0	4.0–4.0	2.3	1.6–3.1	0.040**

Association index (AI), parsimony score (PS), and monophyletic clade (MC) and their significance. While AI and PS indices test for the overall phylogeny and all the characters at once, MC is drawn to specifically quantify the phylogenetic signal for each specific character (occurrence of ciguatera contamination). Asterisk indicates significant values (*: p≤0.1; **: p≤0.05).

## Discussion

### Systematics and taxonomy within Epinephelidae

As in Craig and Hastings [37] (but contradicted by Craig et al. [32], because of the absence of morphological synapomorphy), our result show the inclusion of *Epinephelus leucogrammicus* and *E. altivelis* (previous combinations *Anyperodon leucogrammicus* and *Cromileptes altivelis*) within *Epinephelus* but also the inclusion of *Cephalopholis rogaa* and *C. colonus* within *Cephalopholis* (previous combinations *Aethaloperca rogaa* and *Paranthias colonus*).

Craig and Hastings [37] included in the genus *Mycteroperca* several species usually considered as members of *Epinephelus*. Based on our results, we confirm these new combinations. In addition, we proposed to transfer *E. poecilonotus* (Temminck and Schlegel, 1842) in *Mycteroperca* as *M*. *poecilonota*.

They also observed a monophyletic lineage distinct from the remaining species of *Epinephelus* and from *Mycteroperca*, for which they resurrected the oldest available generic name *Hyporthodus*. Our topologies corroborate this lineage, which includes at least two species, *H. septemfasciatus* and *H. haifensis* (previously *Epinephelus haifensis*).

The relationships between *Belonoperca chabanaudi*, *Diploprion bifasciatum* and *Aulacocephalus temminckii* in Figure S1 question the monophyly of the Diploprionini. In Figure 1A where *A*. *temminckii* was not represented, *B. chabanaudi* is included in the subfamily Epinephelinae (PP = 0.96, BP_ML_ = 54). *D. bifasciatum* constituted, with a weak support, the sister-group of the Grammistinae with the Liopropominae. Since the support for several deeper clades is low at least for maximum likelihood analyses, we prefer not to discuss further the phylogeny of these groups.

Within Epinephelidae, there is no modification of the subfamilies Grammistinae and Liopropominae. The Epinephelinae now include only twelve genera (minus *Anyperodon*, *Cromileptes* and *Paranthias* vs. 15 previously). In our results (Fig. 1), *Variola* and *Plectropomus*-*Saloptia* are in basal position relative to the other Epinephelinae genera.

### Ciguatera fish poisoning

In the studies on ciguatera reviewed, most were fairly unhelpful to establish a list of ciguatera-affected species with scientific names. Most publications use vernacular names only, and many of these designate several fish species (see [2], [6]). Vernacular names might be important for local communication and consumer warnings. However, scientific names should be systematically associated to them to enhance precision and communication between localities, as vernacular name use varies with geography. Feedback questionnaires [63], [64] were provided to public health and fisheries department staff to collect ciguatera poisoning data and then to put more monitoring and research into place. Traceback investigations of fish associated with outbreaks provide valuable information regarding fishing areas associated with CFP. However, in a weekly report, the Centers for Disease Control and Prevention pointed to limitations in the personal feedbacks [65]; where physician reports were unavailable, the symptoms were based entirely on self-report or second hand reports from family members and may be wrong. Moreover, additional cases might have occurred but were unrecognized because involved physicians were not aware of the need to make an appropriate diagnosis and to report, especially in countries where the public health network is weakly organized.

With the limitations of our current knowledge in mind, 29 among 163 grouper species are considered ciguateric in the world. While there is no correlation between the phylogeny and the currently known ciguatera status of the species, some clades include a higher number of possibly ciguateric species, and might be interesting to investigate in order to determine the ciguateric status of species where it has not been described yet. The recent changes in classification based on the reanalyses of the relationships within Epinephelidae ([37], this study) have an impact on the interpretation of the ciguatera distribution in the genera. Multiple species from the genus *Epinephelus* that were not reported to be ciguateric belong in fact to other genera that have very few ciguateric species (*Hyporthodus* and *Mycteroperca*). As for the genera *Plectropomus* and *Variola*, the species included in our datasets are all considered potentially ciguateric (4 and 2 species respectively). In the three other species contained in the genus *Plectropomus*, only one, *P. oligacanthus*, is ciguateric according to FishBase.

In our study, even if the COI gene gives little support for deeper nodes in phylogenetic analyses, it performs well in Epinephelidae for interspecific relationships, as already observed for other Teleosts and taxonomic groups [66], [67]. Better phylogenetic performance means that even if an identical sequence is not available for identification in the database, the position of the unknown sequence in a phylogenetic analysis can provide information about the genus it belongs to, and which species it is most closely related to. Consequently, samples from species that cannot be readily identified, but fall within these groups in an analysis might best be considered potentially ciguateric when tested for ciguatera case suspicions. This, added to the large dataset already available, and the more stringent guidelines of the Barcode of Life project compared to other sequence databases, makes it a very good choice for identification in the group.

Many questions remain to be answered pertaining to the production and accumulation of ciguatoxins and the subsequent occurrence of ciguatera fish poisoning. For example, why does Ciguatera affect only some species in a given locality, why does Ciguatera not affect species over their whole range? Explanations might be found on the life history traits of groupers. The diet, the location on the reef and the size of grouper species could explain some of these ciguateric patterns. Yet no clear tendency based on these data appears. On the other hand, some species like *Epinephelus macrospilos* or *Cephalopholis miniata* are ciguateric, for example, in New Caledonia (Fig. 2) but not in their whole range. Richlen et al. [68] were some of the first authors to correlate the geographic patterns of fish toxicity with the preponderance of highly toxic strains of *Gambierdiscus* spp. They showed that *G. toxicus* was not a single cosmopolitan species, but instead was a species complex comprised of several distantly related groups co-occurring across geography. Thus the range of *G. toxicus* appears to be much smaller than the range of the fish species, but this also depends on the correctness of our knowledge about the systematics and distribution of the fish species. The presence and relative abundance of the members of this species complex among geographic regions may help explaining patterns of ciguatera toxicity, particularly if differences in physiology and/or toxin-producing capabilities also exist among these groups. In addition, a better understanding of the three groups of ciguatoxins produced by *Gambierdiscus* species will be very useful.

As suggested by several authors, due to recent climate change or human activities, harmful algal blooms (HAB), including *Gambierdiscus* spp. [1], [8], [9] might become more frequent and more geographically widespread. A consequence would be that ciguatera starts affecting fish species in other localities in their range where there was no previously recorded problem, affecting populations that will be not aware of the ciguateric risk of fish consumption. The first reports of consumer illness and detection of ciguatoxic fish from the Canary Islands [55] seems to be consistent with such a geographic expansion of ciguatera. Another recent expansion of dinoflagellates, and subsequent incidences of CFP illness, was recorded in the northern Gulf of Mexico, USA [10]. Of the 29 ciguateric grouper species, 16 have a very large distribution in the Indo-Pacific Ocean. The ranges extend from the East of Africa to the West of America, including the Red Sea. Current distribution areas might help us determine future changes in the prevalence area of ciguatera.

### Barcoding, a useful tool for various applications

As all our knowledge on ciguateric species hinges on correct identification of the species involved in CFP, DNA barcoding is an essential tool in controlling CFP, but also investigating mislabelling of seafood or endangered species monitoring. Fishermen and restaurant owners do not hesitate to sell fish of lower quality under erroneous labelling [16], a problem compounded in groupers by overfishing. Because of the very high price of live groupers, the mislabelling of fishes and the dangers of ciguatera, accurate species identification is part of what makes the quality of the fish meat [16]. The misidentification of several species was largely discussed by Stewart et al. [12]. They encountered nine misidentifications in their molecular analyses, and remarked that eight of the nine misnamed fish contain ciguatera toxins. While the authors did not suggest deliberate substitutions, they wondered about this high rate. Some confusions were not surprising in regard of the similar morphology of the species (Spanish mackerel - *Scomberomorus commerson* and *Scomberomorus queenslandicus* for instance). However, they pointed out that selling the Spanish mackerel – a ciguatera-prone species banned in Platypus Bay, Queensland – as red snapper and swordfish, neither known to be problematic for ciguatera, represents a potential risk of bypassing ciguatera prevention strategies [12].

In such a case, DNA barcoding has proved to be a very useful tool to quickly and easily identify seafood species sold on fish markets or on restaurants, thus helping to avoid at least some of the risky species. The mislabelling studies have also had a positive effect on at least some parts of the retail sector [69]. Moreover, meal remnants in CFP cases often cannot be identified without molecular tools, where proper identification is critical for improving the ciguatera affected species list and warnings. DNA barcoding-based traceability procedures were implemented in several U.S. state and federal laboratories [65]. As the symptoms appear in minutes to hours after the ingestion of contaminated seafood [13], recovery of samples for sequencing is possible, at least in some cases. For most types of cooked seafood, the full barcode can be obtained. For severely degraded or heavily processed products (e.g. canned), the sequencing of shorter sequences (i.e. Mini barcodes, see [70]), also works for identification.

### The key role of the public databases

For DNA barcoding to be a useful tool for identifying species, it needs complete (or at least large) and reliable reference sequences available in public databases. For an example of both the usefulness of barcoding methods and the limits of the available databases, a sample of fish tissue collected at the fish market in Nouméa (New Caledonia) showed 100% identity in the databases with those of two distinct species. Both are deep-sea species, as was the grouper from the market according to the fishmongers. *H. ergastularius* is not recorded from New Caledonia, but *H. octofasciatus* was recorded, sometimes under other names [71]. Unfortunately, identical sequences for *H. ergastularius* and *H. octofasciatus* in databases preclude an identification of this sample. Currently, available grouper sequences contain several cases of misidentification both in GenBank and in BOLD. The numerous grey-brown species with dots (*E. tauvina*, *E. akaara*, *E. diacanthus*, *E. amblycephalus*, *E. longispinis*, *E. sexfasciatus* and *E. macrospilos*) are particularly affected, with the additional problem that young specimens of some species resemble adults of smaller species. This is not an isolated case. According to Vilgalys [72] up to 20% of the named sequences in public databases may be misidentified. Hassanin et al. [73] suggested to annotate database sequences through an additional “external expertise” field, and there is indeed a possibility to add comments to data in BOLD. However, a lot remains to be done before all available identifications can be trusted, even with the additional geographical and voucher information required by BOLD. There is an ongoing effort by the BOLD crew to flag dubious sequences. They developed a system to grade the level of reliability of the identification in BOLD [74], but it has not been applied to all sequences yet. In the end, a morphological study of the voucher specimen remains necessary, but this is not practical when fast identification is needed such as for ciguateric sample identifications. While currently neither reliability nor comprehensivity are at hand for Epinephelinae sequences, our study has added 47 COI sequences for carefully identified and vouchered specimens.

## Supporting Information

Figure S1 **Phylogenetic relationships within Epinephelidae.** Bayesian inference phylogram obtained from phylogenetic analyses of the COI under the GTR + I + Γ model. Values at nodes indicate Bayesian posterior probabilities (PP) and maximum likelihood bootstrap percentages (BP). Black circles indicate nodes supported by posterior probability ≥95% and ML bootstrap probability ≥75%.
(TIF)
