# Supplementary figures and images for: A Phylogenetic Re-Analysis of Groupers with Applications for Ciguatera Fish Poisoning
Source: PLoS One. 2014 Aug 5;9(8):e98198. doi: 10.1371/journal.pone.0098198 (PMC4122351; doi:10.1371/journal.pone.0098198)

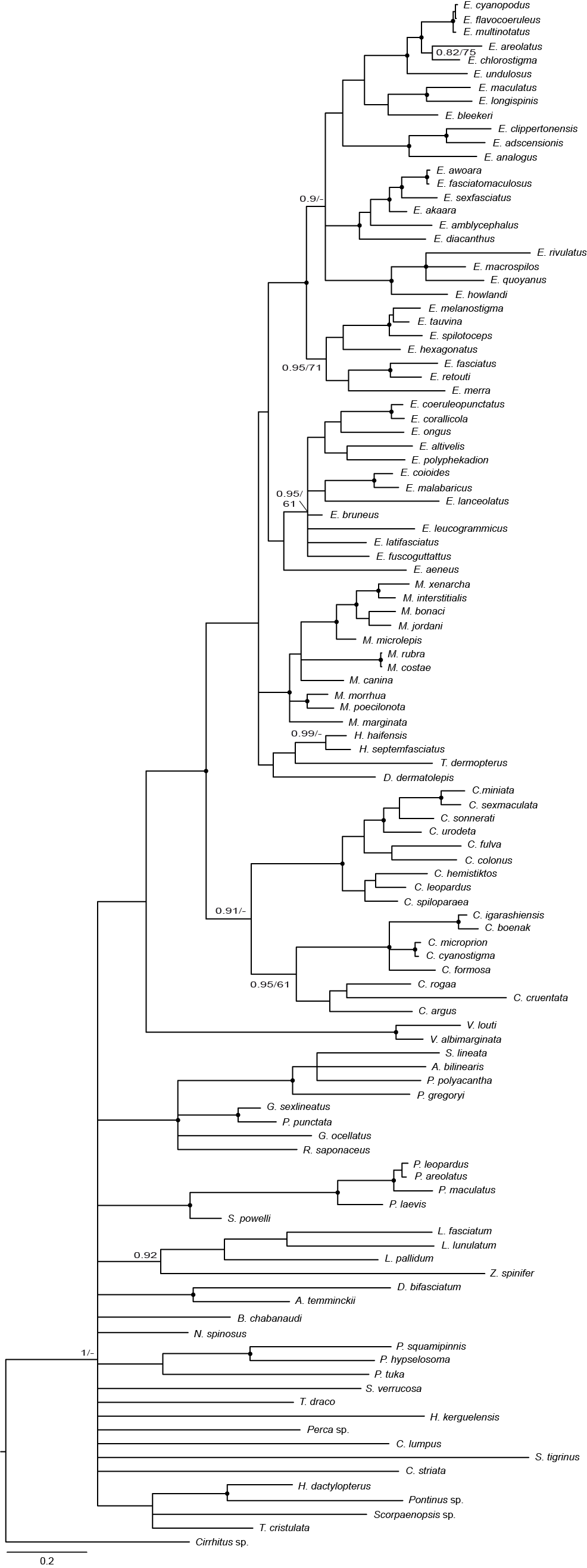

Supplement: Figure S1 — Phylogenetic relationships within Epinephelidae. Bayesian inference phylogram obtained from phylogenetic analyses of the COI under the GTR + I + Γ model. Values at nodes indicate Bayesian posterior probabilities (PP) and maximum likelihood bootstrap percentages (BP). Black circles indicate nodes supported by posterior probability ≥95% and ML bootstrap probability ≥75%. (TIF) [file pone.0098198.s001.tif]
